# Supplementary material for: Examination of factors associated with the temporal stability assessment of crash severity by using generalised linear model—A case study
Source: PLoS One. 2024 Apr 19;19(4):e0299094. doi: 10.1371/journal.pone.0299094 (PMC11029646; doi:10.1371/journal.pone.0299094)
Supplement: S1 Table — (DOCX) [file pone.0299094.s001.docx]

**S1 Table. Categorisation of contributory factors (causes of crash) involved in this study.**

| Contributory factors | Subcategories of contributory factors |
| --- | --- |
| Careless driving | Aggressive driving, Drowsy driving, distracted driving, hazardous, negligent, and unsafe driving, improper U-turn, improper crossing, improper stopping, wrong overtaking, failed to maintain a safe distance |
| Vehicular faults | Mechanical problems, electrical problems, old tires. |
| Others* | Climatic conditions, road conditions, driving under the influence of drugs, fog, poor visibility, pedestrian fault, wrong pedestrian crossing, animal crossing, etc. |
| *As the number of crashes caused by climatic conditions and road conditions are found very less in number, so we merged into “Others” category | |
