# Supplementary material for: Examination of factors associated with the temporal stability assessment of crash severity by using generalised linear model—A case study
Source: PLoS One. 2024 Apr 19;19(4):e0299094. doi: 10.1371/journal.pone.0299094 (PMC11029646; doi:10.1371/journal.pone.0299094)
Supplement: S2 Table — (DOCX) [file pone.0299094.s002.docx]

**S2 Table. Independent variable code and remarks**

| Variable | Remarks |
| --- | --- |
| Crash severity | Continuous variable |
| Day=1 (Monday) | Variable refers to Monday. |
| Day=2 (Tuesday) | Variable refers to Tuesday. |
| Day=3 (Wednesday) | Variable refers to Wednesday |
| Day=4 (Thursday) | Variable refers to Thursday |
| Day=5 (Friday) | Variable refers to Friday |
| Day=6 (Saturday) | Variable refers to Saturday |
| Day=7 (Sunday) | Variable refers to Sunday (reference variable) |
| Beat=1 to Beat-11 | Variable refers to Beats from 1 to 11 having Beat-11 as reference variable |
| Causes of crash=1 (Careless driving) | Variable refers to careless driving |
| Causes of crash=2 (Vehicle conditions) | Variable refers to vehicle conditions |
| Crash cause=3 (Other factors) | Variable refers to other factors (reference variable) |
| Vehicle involved=1 (3W) | Variable refers to 3W vehicles |
| Vehicle involved=2 (LTV) | Variable refers to LTV |
| Vehicle involved=3 (HTV) | Variable refers to HTV (reference variable) |
| Season=1 (Hot) | Variable refers to Hot season having others season as reference variable |
| Season 2 (Others) | Variable refers to all other seasons (reference variable) |
| Time =1 (Daytime) | Variable refers to the daytime |
| Time =2 (Nighttime) | Variable refers to the night-time (reference variable) |
