# Supplementary material for: Examination of factors associated with the temporal stability assessment of crash severity by using generalised linear model—A case study
Source: PLoS One. 2024 Apr 19;19(4):e0299094. doi: 10.1371/journal.pone.0299094 (PMC11029646; doi:10.1371/journal.pone.0299094)
Supplement: Attachment — Submitted filename: Response to Reviewers.docx [file pone.0299094.s003.docx]

January 18, 2023

**Yajie Zou**

**Academic Editor**

**PLOS ONE Journal**

Dear Editor,

I would like to submit the revised manuscript (Manuscript ID PONE-D-23-42042) based on the reviewers’ comments in PLOS ONE. The manuscript is entitled ***"Examination of factors associated with the temporal stability assessment of crash severity by using Generalised Linear Model-A case study".*** We highly appreciate the careful review of our manuscript and the valuable comments and suggestions, which are indispensable for improving the quality of the manuscript. We have carefully read all the suggestions and have given the point-to-point response to each comment. Please note that our responses are in the different colored highlighted in the revised manuscript.

| **Colors used in the revised manuscript** | |
| --- | --- |
| Yellow | Yellow highlight colour is used for the addressing of Journal’s requirements |
| Turquoise | Turquoise highlight colour is used for the addressing of concerns raised by Reviewer # 01 |
| Pink | Pink highlight colour is used for the addressing of concerns raised by Reviewer # 02 |
| Bright Green | Bright green colour is used for the addressing of common concerns raised by both reviewers |
| Black | The same text with the previous manuscript is written in black color |

Sincerely,

Dr Muhammad Hussain

Research Fellow, Western Australian Centre for Road Safety Research

School of Psychological Science • M304, Perth WA 6009 Australia

T +61 0434315925 • muhammad.hussain@uwa.edu.au • Web [www.uwa.edu.au/wa-centre-for-road-safety-research](http://www.uwa.edu.au/wa-centre-for-road-safety-research)

The following summarizes our point-to-point responses to the reviewers’ comments.

**Journal requirement**

1. Please ensure that your manuscript meets PLOS ONE's style requirements, including those for file naming. The PLOS ONE style templates can be found at
   [https://journals](https://journals/).plos.org/plosone/s/file?id=wjVg/PLOSOne_formatting_sample_main_body.pdf and 
   <https://journals.plos.org/plosone/s/file?id=ba62/PLOSOne_formatting_sample_title_authors_affiliations.pdf>

**Response:** We modified our manuscript according to PLOS ONE's style requirements.

2. Please note that funding information should not appear in any section or other areas of your manuscript. We will only publish funding information present in the Funding Statement section of the online submission form. Please remove any funding-related text from the manuscript.

**Response:** The suggestion has been incorporated.

3. We note that the grant information you provided in the ‘Funding Information’ and ‘Financial Disclosure’ sections do not match.

**Response:** We have improved the funding information.

4. When you resubmit, please ensure that you provide the correct grant numbers for the awards you received for your study in the ‘Funding Information’ section.

**Response:** The suggestion has been incorporated.

5. Thank you for stating the following in your Competing Interests section:
   "No competing interest". Please complete your Competing Interests on the online submission form to state any Competing Interests.

**Response:** The suggestion has been incorporated.

6. We note that Figure 3 in your submission contain map/satellite images which may be copyrighted. All PLOS content is published under the Creative Commons Attribution License (CC BY 4.0), which means that the manuscript, images, and Supporting Information files will be freely available online, and any third party is permitted to access, download, copy, distribute, and use these materials in any way, even commercially, with proper attribution. For these reasons, we cannot publish previously copyrighted maps or satellite images created using proprietary data, such as Google software (Google Maps, Street View, and Earth). For more information, see our copyright guidelines: <http://journals.plos.org/plosone/s/licenses-and-copyright>.

**Response:** Figure 3 is not copyrighted.

**Reviewer 1**

The authors are suggested to carefully address the comments below:

1. The paper writing should be significantly improved.

Response: Thank you for your suggestion. We tried our best to make this manuscript according to your valuable suggestions and make this study target oriented. We paid more attention to the paper writing and double-checked all the sentences and grammar structure in our manuscript and rectified accordingly. For improvement of language, we used Grammarly premium editing services to enhance the English language of the manuscript.

(Please refer to text in the revised manuscript highlighted by Turquoise color)

2. In the literature review section, many recent methods for analyzing crash severity are missing.

Response: Thank you for your suggestion. In the revised manuscript, the recent methods for analysing crash severity are incorporated.

(Please refer to lines 71-82)

3. When modeling the effect of different variables on crash, different coefficients are estimated for the same variable across different years. To overcome the model uncertainty, the authors are suggested review the Bayesian model averaging approach which can consider the effect of different variables. For example, see: Application of Bayesian model averaging for modeling time headway distribution. Physica A: Statistical Mechanics and Its Applications, 620, 128747. Improving transferability of safety performance functions by Bayesian model averaging. Transportation research record, 2280(1), 162-172. This point should be discussed as the future work.

Response: The suggestion has been incorporated.

(Please refer to lines 364-374)

4. Why do the authors use the GLM model in this study? How about other modeling approaches?

Response: Thank you for raising an important concern. We added the previous techniques used in crash severity analysis and why we used GLM in this study.

(Please refer to lines 71-82) {the common concern by both reviewers}

&

(Please refer to lines 164-168)

5. It seems that Year 2013 and Year 2017 do not have sufficient data observations for the analysis in this study.

Response: Thank you for your comment. The percentage of fatal crashes in total crash data for the years 2013 and 2017 is 15.8% and 13.3%, respectively. This data observation holds significance for the analysis. Furthermore, the road crash data obtained represents five years of road crashes, from 2013 to 2017. More recent data was not available in a consistent format at the time of this research. Therefore, we remained sticked with 2013-2017 crash data.

6. Why not aggregate all observations into one single dataset and then apply the analysis approach?

Response: Thank you for your comment. The objective of this paper is to explore the temporal stability of the road crashes. And the temporal stability of crashes refers to how consistent or stable the occurrence and characteristics of crashes remain over time. Therefore, it was necessary to aggregate all observations based on each year. This helps identify if crash rates or severity follow consistent trends over time or if they vary, which can be crucial for implementing effective road safety measures and policies.

**Reviewer 2**

1. The total sample size should be explained in Table 1.

Response: Thank you for the suggestion. The Table 1 is now updated.

(For details please refer to Table 1)

2. Why do the authors build models for different years? Does single year data contain enough samples for regression analysis?

Response: Thank you for asking the crucial question. This paper aims to investigate the temporal stability of road crashes, assessing the consistency and stability of their occurrence and characteristics over time. To achieve this, models were constructed for different years instead of a single regression model. The objective is to determine whether crash rates and severity exhibit consistent trends over time or if variations occur. This information is crucial for the implementation of effective road safety measures and policies.

3. For the regression results, some variables are insignificant.

Response: Yes, for the regression results, different variables for different years are found to be insignificant. For instance, Table 3 to Table 7, the values of all the significant variables are written in bold letter. The significant variables for each year are also mentioned in Table 8.

(For details please refer to Table 3, 4, 5, 6, 7 and 8)

4. For Table 8, how to explain the difference in the modeling results?

Response: Table 8 provides a list of variables used in the modeling results for each year, with corresponding values from Tables 3 to 7. Significant variables are marked with a ✓, while non-significant ones are marked with a ✗.

5. What are the practical contributions of this study?

Response: The results of this study could aid decision-makers in identifying the factors that consistently contribute to an increased risk of crash severity of road crashes, allowing for targeted allocation of resources to mitigate the effects of these factors. The results of this research offer specific quantitative measures that can aid in assessing the practicality of implementing various strategies aimed at reducing the risk of crash severity of road crashes in Pakistani drivers. Considering the existing findings, some immediate road safety countermeasures are hereby suggested to minimise the life losses in road safety.

(For details please refer to Lines 375-383)

6. The traffic risk can be reduced with the development of ADAS, this point should also be discussed in the study. And some recent studies about ADAS and traffic risk should be reviewed. For example, see: Spatiotemporal Interaction Pattern Recognition and Risk Evolution Analysis During Lane Changes. IEEE Transactions on Intelligent Transportation Systems. Traffic Risk Assessment Based on Warning Data. Journal of Advanced Transportation, 2022.

Response: Thank you very much for pointing out the relevant issues which could further enhance the strength and quality of the paper. We have incorporated the importance of ADAS in reducing traffic risks and cited the suggested literature in our paper to improve the quality and significance of this study in order to improve the road safety of countries having similar traffic problems.

(For details please refer to Lines 386-393)
